# Microtubule Anchoring: Attaching Dynamic Polymers to Cellular Structures

**DOI:** 10.3389/fcell.2022.867870

**Published:** 2022-03-03

**Authors:** Chithran Vineethakumari, Jens Lüders

**Affiliations:** Institute for Research in Biomedicine (IRB Barcelona), The Barcelona Institute of Science and Technology, Barcelona, Spain

**Keywords:** MTOC, anchoring, microtubule, centrosome, nucleation

## Abstract

Microtubules are dynamic, filamentous polymers composed of α- and β-tubulin. Arrays of microtubules that have a specific polarity and distribution mediate essential processes such as intracellular transport and mitotic chromosome segregation. Microtubule arrays are generated with the help of microtubule organizing centers (MTOC). MTOCs typically combine two principal activities, the *de novo* formation of microtubules, termed nucleation, and the immobilization of one of the two ends of microtubules, termed anchoring. Nucleation is mediated by the γ-tubulin ring complex (γTuRC), which, in cooperation with its recruitment and activation factors, provides a template for α- and β-tubulin assembly, facilitating formation of microtubule polymer. In contrast, the molecules and mechanisms that anchor newly formed microtubules at MTOCs are less well characterized. Here we discuss the mechanistic challenges underlying microtubule anchoring, how this is linked with the molecular activities of known and proposed anchoring factors, and what consequences defective microtubule anchoring has at the cellular and organismal level.

## Introduction

Microtubules, elongated, cylindrical polymers assembled from heterodimers of α- and β-tubulin, are major elements of the cytoskeleton that mediate a wide range of functions in cycling as well as post-mitotic, differentiated cells. The orientation of tubulin dimers within the microtubule lattice provides microtubules with an intrinsic polarity, exposing β-tubulin at the “plus-end” and α-tubulin at the “minus-end” (Alushin et al., 2014). Microtubule polarity is recognized by motor proteins to allow directed transport. Whereas most kinesins are plus-end-directed, the dynein motor and a few kinesins move towards the minus-end. Other proteins interact specifically with either of the two ends to modulate its dynamic behaviour through stabilization or destabilization (Akhmanova and Steinmetz, 2015). To function efficiently and to fulfil the specific needs of different cell types and cell cycle stages, microtubules are arranged into various types of arrays. These arrays differ in shape and distribution and may contain microtubules of uniform or mixed polarity and of variable dynamicity (Sallee and Feldman, 2021). To generate different types of microtubule arrays, cells employ microtubule organizing centers (MTOCs) (Sanchez and Feldman, 2017; Wu and Akhmanova, 2017; Paz and Lüders, 2018). MTOCs can be assembled at the cytoplasmic surfaces of various organelles. The best-known example is the centrosomal MTOC, which is assembled around centrioles, but other, typically membrane-bound organelles such as the Golgi or the nuclear envelope, can also acquire MTOC activity.

A key component of most MTOCs is the microtubule nucleator γTuRC, which allows generation of new microtubules. Mimicking the structure of a microtubule in cross-section, the circular, helical arrangement of γ-tubulins in the γTuRC provides docking sites for tubulin heterodimers, promoting their lateral interactions and facilitating polymer formation (Moritz et al., 2000; Consolati et al., 2020; Liu et al., 2020; Thawani et al., 2020; Wieczorek et al., 2020; Zimmermann et al., 2020). Since soluble native and recombinant γTuRCs are intrinsically asymmetric and do not perfectly match the microtubule symmetry, they likely require activation, for example through a conformational change (Kollman et al., 2015). Alternatively, nucleation may be stimulated by cofactors that interact with and stabilize tubulin assembly intermediates on γTuRC such as members of the XMAP215 family of tubulin polymerases (Wieczorek et al., 2015; Flor-Parra et al., 2018; Gunzelmann et al., 2018; Thawani et al., 2018).

Apart from nucleation, the second, possibly most important activity of MTOCs is their ability to anchor microtubules. While most MTOCs also nucleate microtubules, MTOC activity could, in principle, also be carried out without nucleation, by capturing and anchoring of microtubules that were nucleated elsewhere. Indeed, it is the anchoring of microtubules at MTOCs that ultimately confers specific polarity and shape to microtubule arrays (Sanchez and Feldman, 2017; Wu and Akhmanova, 2017; Paz and Lüders, 2018). Several anchoring factors have been identified (Table 1), but in most cases their molecular functions are unclear and a mechanistic picture is still missing.

**TABLE 1:** Major anchoring factors. Factors reported to be involved in anchoring microtubules at different MTCOs. Factors that likely affect anchoring more indirectly were not included. For each anchoring factor, identified by both the common name and organism-specific name, we indicate the ability to directly bind microtubules, the proposed role in anchoring, and the domains/regions involved in these functions. Question marks indicate cases where experimental data is not available. Abbreviations used: Sc–*S. cerevisiae*, Sp–*S. pompe*, At–*A. thaliana*, Hs–*H. sapiens*, Dm–*D. melanogaster*, Ce–*C. elegans*, SDA–Subdistal appendages, CC–Coiled coil.

Protein	Organism	Anchoring site	Microtubule binding	Role in anchoring	References
Stu2	Sc	Spindle pole body (SPB)	Direct binding (TOG domains; C-terminal region)	Minus-end stabilization; SPB and γ-tubulin complex binding (C-term. region)	Usui et al. (2003); Al-Bassam et al. (2006)
Spc72	Sc	SPB	?	Anchoring γ-tubulin complex and Stu2 (N-term. region)	Usui et al. (2003)
Pkl1	Sp	SPB (through Msd1)	Direct binding (motor domain)	Anchoring γ-tubulin complex	Yukawa et al. (2015)
Wdr8	Sp	SPB (through Msd1)	?	Anchoring γ-tubulin complex	Yukawa et al. (2015)
	At	Cortical microtubule array branch points	?	Minus-end stabilization at branch site	Yagi et al. (2021)
	Hs	Centrosome	?	Forms anchoring complex with Msd1; astral microtubule organization; spindle positioning	Hori et al. (2015)
Msd1	Sp	SPB	through Pkl1	Anchoring γ-tubulin complex	Yukawa et al. (2015)
	At	Cortical microtubule array branch points	?	Minus-end stabilization at branch site	Yagi et al. (2021)
	Hs (SSX2IP)	Centrosome	?	Anchoring γTuRC to PCM	Hori et al. (2014), Hori et al. (2015)
NEDD1	Hs	Centrosome	?	Anchoring γTuRC; centrosome binding (WD40 repeats); γTuRC binding (C-term. region)	Haren et al. (2006), Lüders et al. (2006), Manning et al. (2010), Muroyama et al. (2016)
FSD1	Hs	Centrosome (centriole central region)	Direct binding (SPRY domain)	Anchoring minus-ends (CC region for localization)	Tu et al. (2018)
Dynein complex	Hs	Centrosome; apical membrane	Direct binding (motor domain; CAP-Gly domain of p150^*glued*^ subunit)	Connecting microtubules to anchoring adapters	Quintyne et al. (1999), Askham et al. (2002), Culver-Hanlon et al. (2006), Kodani et al. (2013), Goldspink et al. (2017)
Ninein	Hs (NIN)Dm (Bsg25D)	Centrosome (SDAs, proximal end);	?	Anchoring γTuRC (N-term. region); dynein adapter (multiple CC regions)	Mogensen et al. (2000), Delgehyr et al. (2005), Moss et al. (2007), Kodani et al. (2013), Goldspink et al. (2017), Rosen et al. (2019)
		apical membrane; nuclear envelope			
	Ce (NOCA-1)	Apical surface	?		Wang et al. (2015)
CAMSAPs	Hs	Centrosome;	Minus-end specific binding (CKK domain)	Minus-end stabilisation; interaction with other anchoring factors (C-term. CC region for localization)	Goodwin and Vale (2010) Jiang et al. (2014), Nashchekin et al. (2016), Toya et al. (2016), Wu et al. (2016), Yang et al. (2017)
	Dm (Patronin)	apical membrane;			
	Ce (PTRN-1)	Golgi			
NDEL1	Hs	Centrosome	?	Dynein regulator (C-terminal region)	Guo et al. (2006)
EB1, EB3	Hs	Centrosome;	Direct end binding (CH domain)	Connecting MTs to anchoring adapters and dynactin complex	Askham et al. (2002), Louie et al. (2004), Yan et al. (2006), Yang et al. (2017)
		Golgi			
CAP350, FOP	Hs	Centrosome	?	Possibly docking EB1 at the centrosome, localisation of FSD1	Yan et al. (2006)
	
AKAP9 (AKAP450)	Hs	Centrosome;	?	Scaffold for MTOC assembly	Wu et al. (2016)
		Golgi			
Myomegalin (MMG)	Hs	Golgi	?	Anchoring CAMSAP bound to minus-ends (N-terminal region)	Wu et al. (2016)
Spectraplakin	Hs (ACF7)	Apical membrane	Direct binding (GAR domain)	Localization and anchoring of CAMSAP3-bound minus-ends; CAMSAP3 binding (spectrin repeat region); actin binding (CH domains)	Leung et al. (1999), Wu et al. (2008), Nashchekin et al. (2016), Noordstra et al. (2016)
	Dm (Shot)				
CLIP170	Hs	Apical membrane	Direct binding (CAP-Gly domains)	Ninein deployment	Folker et al. (2005), Ligon et al. (2006), Goldspink et al. (2017)
IQGAP1	Hs	Apical membrane	?	Ninein deployment	Goldspink et al. (2017)
RAC1	Hs	Apical membrane	?	Ninein deployment	Goldspink et al. (2017)
Piopio	Dm	Apical membrane	?	MTOC assembly	Brodu et al. (2010)

Here, we focus on the anchoring of microtubules at MTOCs and discuss our current knowledge regarding the molecules and mechanisms involved in this process. For an in-depth discussion of MTOCs and associated microtubule nucleation, we refer the reader to several recent reviews (Lee and Liu, 2019; Valenzuela et al., 2020; Wilkes and Moore, 2020; Lüders, 2021; Sallee and Feldman, 2021). We begin, by outlining conceptually how anchoring of microtubules may be achieved, and then, using various types of MTOCs and anchoring factors in different organisms as examples, discuss how available evidence supports these concepts. Finally, we highlight how defective microtubule anchoring impairs the microtubule cytoskeleton and may impair organismal development.

## Molecular Requirements for Microtubule Anchoring

**FIGURE 1:**
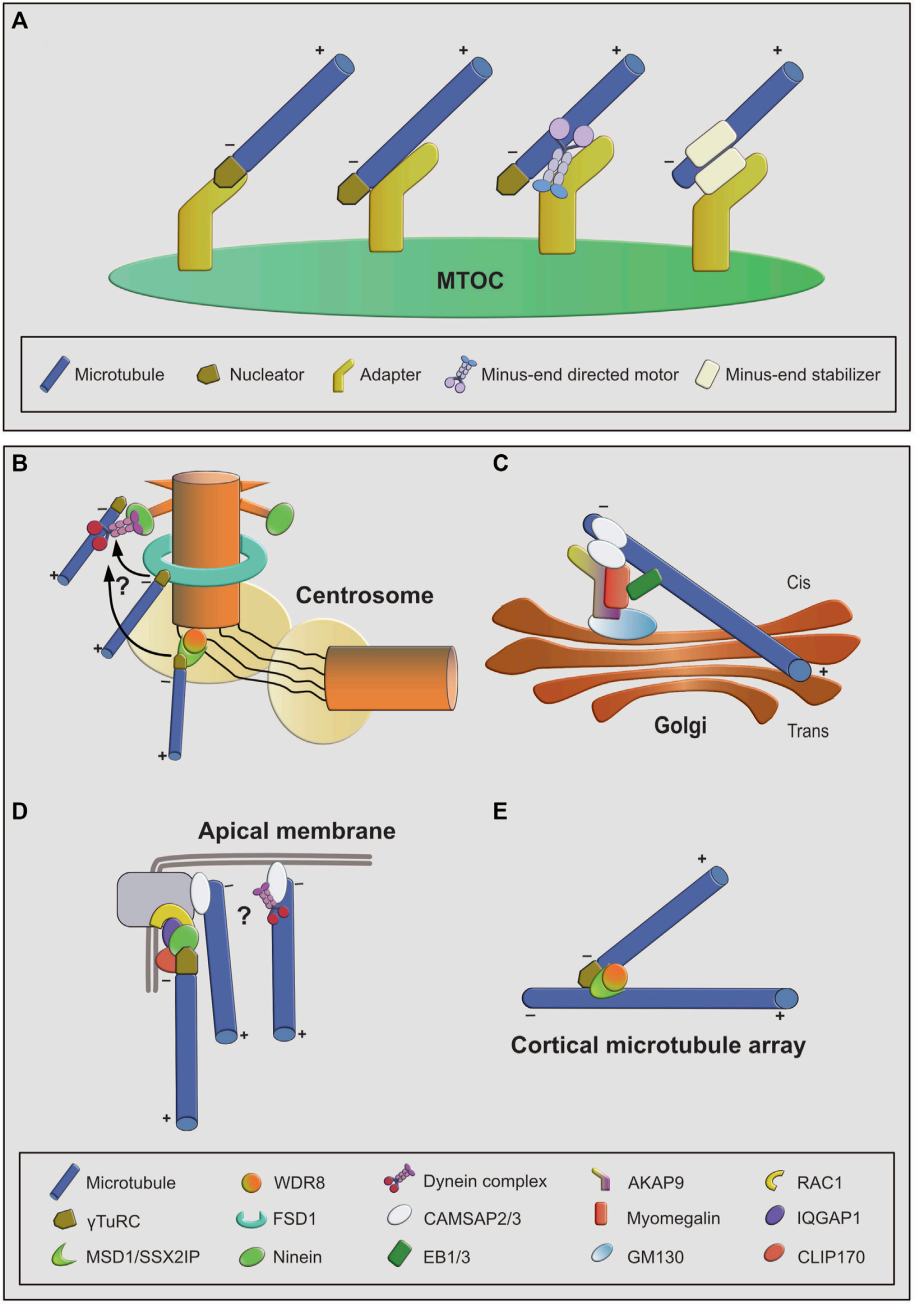
Overview of microtubule anchoring sites and mechanisms. **(A)**: Conceptual overview of mechanisms by which microtubules can be anchored at MTOCs. From left to right: first, the nucleator may be part of the anchoring complex as a stabilizing minus-end cap. Anchoring to the MTOC may be achieved through an MTOC-bound adapter that interacts with the minus-end cap or with the microtubule lattice. Lattice interaction could be direct or indirect via a minus-end directed motor. Second, the nucleator may not be part of the anchoring complex. In this case anchoring is facilitated by an adapter protein interacting with a minus-end-bound, stabilizing protein. **(B–E)**: Examples of MTOCs and associated anchoring factors. **(B)**: At the interphase centrosome anchoring to the mother centriole is achieved through multiple mechanisms, involving ninein-dynein at the subdistal appendages, FSD1 in the central region, and MSD1-WDR8 in the proximal/PCM region. FSD1-and MSD1-mediated anchoring may be transient and minus-ends may be transferred to subdistal appendages. **(C)**: Anchoring at cis-Golgi membranes involves the AKAP9-myomegalin complex as adapter and CAMSAP2 as stabilizer at the microtubule minus-end that connects it to the adapter complex. EB proteins provide an additional way of connecting microtubules to the adapter complex through myomegalin. **(D):** At apical junction complexes and membranes in epithelial cells both ninein- and CAMSAP-mediated anchoring mechanisms may act in parallel. **(E):** At branch points on plant cortical microtubules, MSD1-WDR8 complexes stabilize and anchor the minus-ends of newly nucleated microtubule branches to the lattice of the pre-existing microtubules.

There are two basic requirements to be fulfilled by microtubule anchoring factors (Figure 1A). The anchoring proteins or protein complexes have to bind microtubules and, at the same time, interact with an MTOC. This linkage needs to be not only robust but also flexible, to resist the variable mechanical forces that act on microtubules as they extend away from the MTOC and serve as tracks for motor proteins. Importantly, to allow arrangement of microtubules with a specific orientation, binding of the anchoring factor to the microtubule needs to occur specifically at only one of the two ends. An additional challenge is the dynamic nature of microtubules. In microtubules assembled from pure tubulin *in vitro*, both minus- and plus-ends are dynamic and can undergo phases of growth and shrinkage. Thus, anchoring of microtubules likely involves stabilization and inhibition of microtubule end dynamics (Hendershott and Vale, 2014; Jiang et al., 2014; Akhmanova and Steinmetz, 2015). In the case of microtubules that are nucleated by γTuRC, the nucleator may form a stabilizing cap at their minus-end (Wiese and Zheng, 2000), leaving only the plus-end free to grow or shrink. This is consistent with the observation that anchoring of microtubules to MTOCs typically occurs via their minus-ends, whereas the plus-ends extend away from it and are more dynamic. Assuming that γTuRC remains bound to the minus-ends of newly nucleated microtubules, the nucleator itself could also provide anchoring function, as observed in reconstitution assays *in vitro* (Consolati et al., 2020). However, it seems that in cells anchoring usually involves additional factors (Figures 1B–E).

## Microtubule Anchoring Factors and Mechanisms

### Anchoring in Cycling Cells With a Central MTOC

A relatively simple microtubule network is found in budding yeast, where microtubules are organized by the spindle pole body (SPB), a single MTOC that is equivalent to the centrosome. Here anchoring of cytoplasmic microtubules was shown to involve ternary complexes composed of the SPB-bound adapter Spc72, the γ-tubulin-containing nucleation complex, and Stu2, a member of the XMAP215 family that also functions as nucleation stimulator (Usui et al., 2003; Gunzelmann et al., 2018). Following nucleation at the SPB, minus-ends are capped by γ-tubulin complexes and Stu2 may simultaneously interact with the nucleation complex and with the proximal wall of the newly nucleated microtubule (Usui et al., 2003). Similarly, in fission yeast γ-tubulin complexes cooperate with the XMAP215 homolog Alp14 to nucleate microtubules from the SPB and from the nuclear envelope, a second interphase MTOC, but a role of Alp14 in anchoring has not been described (Flor-Parra et al., 2018; Liu et al., 2019). Specifically during mitosis minus-ends of mitotic spindle microtubules are anchored through the coiled-coil protein Msd1 (Toya et al., 2007). Msd1 functions as part of a ternary complex with two other proteins, Wdr8 and the minus end-directed kinesin-14 motor Pkl1 (Yukawa et al., 2015). Minus-end-directed motor activity of Pkl1 is used to transport Msd1 and Wdr8 towards the SPB, where the ternary complex interacts with γ-tubulin complexes and promotes minus-end anchoring. Interestingly, Pkl1, artificially tethered to the SPB, provided partial anchoring function, even in the absence of Msd1-Wdr8 and using a motor-defective rigor mutant of Pkl1 (Yukawa et al., 2015). One could speculate that in this scenario mutant Pkl1 may still be able to provide the two basic functions of anchoring factors outline above: localization to the SPB and interaction with microtubules. The role of Msd1 in anchoring is conserved. Human MSD1, also known as SSX2IP, was shown to interact with the nucleator γTuRC and promote microtubule anchoring at the centrosome, both in interphase and mitosis (Bärenz et al., 2013; Hori et al., 2014, 2015). Interestingly, at the centrosome MSD1 partially colocalizes with γTuRC, but does not colocalize with ninein/NIN, an established anchoring factor and component of subdistal appendages, structures specific to the mother centriole that have been implicated in microtubule anchoring. Moreover, a C-terminal MSD1 fragment that was sufficient to interact with γTuRC provides anchoring activity when artificially tethered to the pericentriolar material (PCM) in the proximal part of centrioles (Hori et al., 2014). This suggests that microtubules may be anchored not only at subdistal appendages and that anchoring could be coupled with nucleation. However, while human MSD1 also interacts with WDR8 (Hori et al., 2015), the molecular basis of its anchoring activity has not been revealed.

Anchoring of microtubules at the vertebrate centrosome involves subdistal appendages and the activity of the subdistal appendage protein ninein (Bouckson-Castaing et al., 1996; Mogensen et al., 2000; Dammermann and Merdes, 2002; Ou et al., 2002; Delgehyr et al., 2005; Lin et al., 2006). Several proteins described to affect anchoring may do so through altering subdistal appendage structure and/or ninein recruitment (Ibi et al., 2011; Kodani et al., 2013; Huang et al., 2017). Anchoring at subdistal appendages would imply that microtubules nucleated by γTuRC in the more proximally located PCM, which is considered to be the main nucleation site, would be transferred to the subdistal appendages for stable anchoring, possibly with γTuRC as a stabilizing minus-end cap (Delgehyr et al., 2005; Hori et al., 2014). However, γTuRC localizes not only to the PCM and there is some evidence that nucleation may also occur directly at subdistal appendages (Schweizer and Lüders, 2021). The finding that γTuRC interacts with ninein would be consistent with both models (Delgehyr et al., 2005). It should also be noted that centrosomal ninein is not restricted to subdistal appendages, but is also present at the proximal ends of both mother and daughter centrioles. The significance of this localization is not entirely clear but it may be related to ninein’s role in centrosome cohesion (Mazo et al., 2016). How does ninein mediate microtubule anchoring? Ninein’s N- and C-terminal regions were shown to mediate γTuRC-binding and centrosome targeting, respectively, but whether ninein can bind microtubules was not investigated (Delgehyr et al., 2005; Lin et al., 2006). However, ninein was shown to bind dynein (Casenghi et al., 2005) and, more recently, to function as dynein activator (Redwine et al., 2017). Dynein has been implicated in the centrosome targeting of several proteins, in some cases in the form of particles known as centriolar satellites (Kubo et al., 1999; Dammermann and Merdes, 2002; Prosser and Pelletier, 2020). In this case, however, dynein’s ability to bind microtubules and move towards their minus-ends may be invoked by centrosome-bound ninein to anchor microtubules. Consistent with this possibility, several studies have linked dynein complexes with centrosomal microtubule anchoring (Quintyne et al., 1999; Guo et al., 2006; Kodani et al., 2013). Such a mechanism would have to ensure that dynein does not run off the microtubule once it has reached its minus-end. Indeed, at least *in vitro*, certain dynein complexes were observed to remain bound and accumulate at minus-ends (McKenney et al., 2014; Soundararajan and Bullock, 2014). Clearly, further work is needed to elucidate the potential cooperation between ninein and dynein in centrosomal minus-end anchoring.

Apart from MSD1 and ninein discussed above, a recent study has revealed another centrosomal protein, FSD1 (also known as MIR1 and GLFND), as microtubule anchoring factor (Tu et al., 2018). A comprehensive analysis showed that a coiled-coil domain at its N-terminus is sufficient for centrosome localization and that the B30.2/SPRY domain in the C-terminal part directly binds to and is required for anchoring of microtubules at the centrosome. Interestingly, FSD1 localizes in a circular fashion around centrioles, similar to subdistal appendage proteins, but positioned more proximally (Tu et al., 2018). Even though FSD1 localizes also around the daughter centriole, it promotes microtubule anchoring only at the mother centriole, pointing at the involvement of additional factors specific to the mother centriole. Notably, FSD1 and ninein are not dependent on each other for their specific localisations (Tu et al., 2018). The data suggest that FSD1, similar to MSD1, either extends the mother centriole-specific microtubule anchoring activity to the central portion of the cylinder or that it may be involved in the transfer of minus ends from proximally located nucleation sites to the subdistal appendage region for stable anchoring. Additional work is needed to clarify this issue.

Some anchoring factors share the ability to interact with γ-tubulin-containing nucleation complexes. This observation may indicate a mechanistic link between nucleation and anchoring and/or that γTuRC has two separate functions. Apart from providing a nucleation template, it may form a cap structure at minus-ends (Wiese and Zheng, 2000) that is used for microtubule anchoring. If so, distinct subpopulations of γTuRC may exist at centrosomes to mediate nucleation and anchoring, respectively. This was recently suggested to be the case in keratinocytes. In contrast to other cell types, where NEDD1 depletion robustly impairs centrosomal nucleation (Haren et al., 2006; Lüders et al., 2006), in keratinocytes nucleation activity is largely dependent on γTuRC in complex with the PCM protein CDK5RAP2, whereas γTuRC associated with NEDD1 is mainly used for anchoring (Muroyama et al., 2016).

Even in the presence of an active centrosome, MTOC activity associated with the Golgi may significantly contribute to microtubule network organization. This activity may be further enhanced when centrosome activity is compromised (Efimov et al., 2007; Miller et al., 2009; Rivero et al., 2009; Wu et al., 2016; Gavilan et al., 2018). CLASPs were initially proposed to provide anchoring function to microtubules associated with the trans-Golgi network (Efimov et al., 2007), but more recent work suggested that they merely function in stabilization (Wu et al., 2016). AKAP9/AKAP450 is a central organizer of the MTOC at the cis-Golgi that recruits both nucleation and anchoring factors. γTuRC is used to nucleate Golgi-associated microtubules but does not seem to remain bound to their minus-ends. Instead, these are bound by the minus-end-stabilizing protein CAMSAP2, and tethered to Golgi membranes *via* myomegalin (Wu et al., 2016). Curiously, end-binding proteins EB1 and EB3, known as plus-end regulators, were shown to participate in tethering microtubules to Golgi membranes (Yang et al., 2017). Importantly, apart from Golgi-nucleated microtubules, CAMSAP2-decorated microtubules from other sites (e.g., nucleated and released from the centrosome) (Keating et al., 1997; Mogensen, 1999; Dong et al., 2017) can be captured and attached to the Golgi MTOC (Jiang et al., 2014; Wu et al., 2016). While the CAMSAP2-mediated minus-end binding mechanism is quite well understood (Atherton et al., 2017), the interplay with myomegalin and EBs for anchoring at the Golgi much less so.

### Anchoring in Differentiated Cells With Distributed MTOCs

In metazoans, during cell differentiation the centrosome frequently loses its role as central microtubule organizer. As a result, in many specialized cell types microtubules are nucleated and anchored at more broadly distributed, non-centrosomal MTOCs (Paz and Lüders, 2018; Sallee and Feldman, 2021). An extreme case are plants, which lack centrioles altogether. In the plant interphase cortical microtubule array, for example, new microtubules are nucleated as branches from the lattice of pre-existing microtubules. Here the conserved Msd1-Wdr8 module was recently shown to anchor and stabilize microtubule minus-ends at the branch sites (Yagi et al., 2021). In addition, the Msd1-Wdr8 complex recruits katanin to the branch site, to allow severing and release of the newly nucleated microtubule branch. These activities are important for proper cortical microtubule array organization (Yagi et al., 2021).

Early work showed that during the differentiation of vertebrate polarized epithelia ninein expression is essential for cell polarization and formation of the apicobasal array of microtubules in these cells. As cells convert their centrosomal microtubule array to an apico-basal array, ninein is released from the centrosome to relocate anchoring function to an apical, non-centrosomal MTOC (Lechler and Fuchs, 2007; Moss et al., 2007; Bellett et al., 2009; Goldspink et al., 2017). In the epidermis this MTOC is formed in association with desmosomes at cell-cell junctions and is mediated by desmoplakin (Lechler and Fuchs, 2007). In columnar epithelial cells, ninein colocalizes with the adherens junction protein β-catenin. During differentiation, ninein associates with microtubules to be deployed at the apical MTOC in a process that depends on the plus-end interactor CLIP170 and cortical IQGAP1 and active Rac1 (Goldspink et al., 2017). Interestingly, once established, maintenance of the apico-basal microtubule array no longer required ninein. Experimental loss of ninein may be compensated for by apically localized CAMSAP2, and the dynactin subunit p150^Glued^ (Goldspink et al., 2017), which has been implicated previously in anchoring at the centrosome (Quintyne et al., 1999; Kodani et al., 2013). Thus, different anchoring factors and mechanisms contribute and provide redundancy to apical anchoring of microtubules.

Apart from the apical membrane in polarized epithelial cells, ninein has also been identified at other non-centrosomal MTOCs, suggesting a broader role in microtubule anchoring. In mammalian multi-nucleated myotubes and in cardiomyocytes, ninein was identified as part of a non-centrosomal MTOC that forms during differentation at the nuclear envelope (Tassin et al., 1985; Bugnard et al., 2005; Srsen et al., 2009; Vergarajauregui et al., 2020; Becker et al., 2021). In muscle cells from *Drosophila* larvae ninein was also found in association with the perinuclear MTOC (Zheng et al., 2016). Later it was shown that in fly embryonic myotubes ninein cooperates with ensconsin/MAP7 in positioning nuclei along the myotube, which is important for muscle function (Rosen et al., 2019). More recently, a nuclear envelope-associated MTOC containing ninein was also described in *Drosophila* fat body cells, a cell type equivalent to liver adipocytes (Zheng et al., 2020). However, a formal demonstration that ninein mediates anchoring of microtubule minus-ends at these sites, is still lacking.

During neuronal differentiation ninein was observed to relocate from centrosomes to the cytoplasm in different neuronal compartments in the form of small granules, but no specific MTOC was identified (Baird et al., 2004; Ohama and Hayashi, 2009). Subsequently, ninein was revealed as a major transcriptional target of Sip1, a regulator of nervous system development. Loss of ninein phenocopied Sip1 deletion, and exogenous ninein expression was shown to rescue Sip1 deletion phenotypes, promoting axonal growth and branching by enhancing microtubule growth and stability (Srivatsa et al., 2015). It remains unclear though, whether these effects are related to a function of ninein in minus-end anchoring.

CAMSAP family members, which are not present in yeast and plants, can specifically recognize and stabilize minus-ends of non-centrosomal microtubules (Meng et al., 2008; Baines et al., 2009; Goodwin and Vale, 2010; Jiang et al., 2014; Atherton et al., 2017). Consistently, CAMSAPs are also associated with non-centrosomal MTOCs. In polarized epithelial cells in flies, worms and mammals, CAMSAP homologs were shown to contribute to the organization of apico-basal microtubule arrays that have their minus-ends anchored at non-centrosomal, apical MTOCs (Meng et al., 2008; Tanaka et al., 2012; Wang et al., 2015; Nashchekin et al., 2016; Ning et al., 2016; Noordstra et al., 2016; Toya et al., 2016). The contribution of CAMSAPs to apical minus-end anchoring may involve their ability to decorate microtubule minus-ends and to interact with spectraplakins that tether microtubules to the cortical actin network (Khanal et al., 2016; Nashchekin et al., 2016; Sanchez et al., 2021). In the larval epidermis in *C. elegans*, the CAMSAP homolog PTRN-1 functions redundantly with NOCA-1, a worm ninein homolog. Whereas NOCA-1 seems to work together with γ-tubulin, PTRN-1 likely stabilizes minus-ends in the absence of γ-tubulin (Wang et al., 2015). Similarly, in *Drosophila* fat body cells, ninein and patronin, the fly CAMSAP, function in parallel in organizing microtubule minus-ends at the nuclear envelope-associated MTOC. This function did not require γ-tubulin, even though it was also present at the nuclear envelope (Zheng et al., 2020). Recent testing by induced degradation of a panel of candidate factors in *C. elegans* embryonic intestinal epithelial cells has confirmed significant redundancy in apical MTOC assembly and anchoring mechanisms (Sallee et al., 2018). A novel type of MTOC that lacked detectable γ-tubulin was recently described within varicosities of the basal process of highly polarized neural progenitors/radial glial cells in the brain (Coquand et al., 2021). CAMSAPs accumulated in the varicosities and knockdown of CAMSAP1/2 reduced microtubule growth from these sites and destabilized the entire basal process. Since the varicosities were positive for trans-Golgi and trans-Golgi-network markers, the microtubule-anchoring structures may be similar to those of the Golgi-associated MTOC (Wu et al., 2016; Coquand et al., 2021).

## Consequences of Microtubule Anchoring Defects

In cycling cells, centrosomal anchoring defects are expected to reduce the fidelity of mitotic spindle assembly, and impair the positioning of spindles, which relies on astral microtubule anchoring around centrosomes at the spindle poles. Anchoring defects at non-centrosomal MTOCs during differentiation, will likely interfere with proper microtubule network remodelling, which is required for the morphological and functional adaptations that cells undergo to carry out specific functions. Indeed, ninein depletion in cultured human cells prevents the organization of a radial, centrosome-centered interphase microtubule array, and causes multipolar spindles in mitosis (Dammermann and Merdes, 2002; Logarinho et al., 2012). In the early fly embryo, maternally provided ninein is required for proper mitotic spindle assembly, but it is not essential at later developmental stages (Kowanda et al., 2016; Zheng et al., 2016). Ninein in neural progenitors of the developing mammalian brain has a role in progenitor interkinetic nuclear migration, asymmetric centrosome inheritance, and progenitor maintenance (Wang et al., 2009; Shinohara et al., 2013). Depletion of the neural progenitor pool by mitotic defects has been shown to cause microcephaly in mouse models of Seckel syndrome, a developmental disorder that is caused by mutations in genes encoding centrosome proteins including ninein (Dauber et al., 2012; Marjanović et al., 2015). Additional work in ninein KO mice has revealed defects in the skin. Ninein loss was found to disrupt correctly oriented progenitor cell divisions and, during epidermal cell differentiation, the formation of non-centrosomal cortical microtubule arrays, impeding desmosome assembly and skin barrier formation. These defects are reminiscent of epidermis defects observed in *C. elegans* NOCA-1 (ninein) and PTRN-1 (CAMSAP) double loss-of-function mutants (Wang et al., 2015).

Several of the factors that contribute to anchoring microtubules at the interphase centrosome have also been implicated in the assembly of primary cilia, surface-exposed signalling organelles that form as an extension of the distal end of the mother centriole. Ciliary defects cause a group of developmental disorders known as ciliopathies. FSD1, ninein and KIF3A promote assembly of the ciliary transition zone, a critical step in ciliogenesis. At least in part this involves the formation and trafficking of centriolar satellites along mother centriole-anchored microtubules (Kubo et al., 1999; Kodani et al., 2013; Tu et al., 2018; Odabasi et al., 2019). Similar observations were made for MSD1, which is required for ciliogenesis in cultured cells and in zebrafish embryos (Hori et al., 2014). Subdistal appendage anchoring of microtubules is also important for proper positioning of cilia, which allows surface exposure of primary cilia (Mazo et al., 2016) and, in the case of motile cilia, coordination of ciliary beating (Kunimoto et al., 2012).

Loss of CAMSAP family members does not seem to affect centrosomes but rather non-centrosomal MTOCs. In flies, cortical patronin helps to define the anterior-posterior axis in the oocyte and, during abdominal epidermis formation, it is required for epithelial remodelling and proper abdomen development (Nashchekin et al., 2016; Panzade and Matis, 2021). Homozygous deletion of CAMSAP3’s microtubule-binding domain in mice resulted in growth defects and, at the cellular level, in mispositioning of organelles. The architecture of polarized intestinal epithelial cells was only mildly affected, consistent with redundancy in apico-basal polarity organization (Toya et al., 2016). Analysis of CAMSAP loss-of-function in invertebrate and vertebrate models has revealed a wide range of phenotypes such as axon and dendrite growth and branching defects, reduced cell survival and organ size, or loss of ciliary motility (Chuang et al., 2014; Marcette et al., 2014; Richardson et al., 2014; Robinson et al., 2020; Liu et al., 2021; Yang and Choi, 2021). However, since CAMSAPs are likely general minus-end stabilizers rather than dedicated anchoring factors, some of these phenotypes may not necessarily result from anchoring defects, but, for example, from an overall reduction in microtubule density in CAMSAP-deficient cells (Jiang et al., 2014).

## Conclusion and Outlook

The increasing interest in microtubule anchoring mechanisms has led to several important discoveries during recent years. This has also been facilitated by the use of invertebrate models such as *Drosophila melanogaster* and *C. elegans*, which are particularly useful for studying non-centrosomal MTOCs in the context of differentiated cells and tissues. The emerging picture is that MTOCs use multiple anchoring factors and mechanisms, often resulting in redundancy. While some mechanisms depend on the nucleator γTuRC, presumably as a stabilizing minus-end cap, others rely on γTuRC-independent anchoring, employing alternative minus-end stabilizers such as CAMSAP family members.

Important open questions are how microtubule minus-end binding is achieved, in particular for anchoring factors that do not directly bind to microtubules, and whether the presence of multiple anchoring mechanisms at a single MTOC simply provides redundancy or, alternatively, may indicate the presence of distinct anchoring sites that are specific for subsets of microtubules (Sallee et al., 2018). For example, dynamic microtubules may be anchored differently than more stable microtubules. This distinction may depend on the nucleation mechanism and site used to generate these microtubules, and may also involve specific post-translation modifications on their lattice (Janke and Magiera, 2020).

One major obstacle in studying minus-end organization at MTOCs is the crowded nature of these areas. Thus, when addressing the above questions, the consequent use of super resolution techniques including expansion microscopy should enable researchers to probe anchoring sites with improved spatial resolution, to dissect single microtubule minus-ends, their post-translational modifications, and their associated molecules.
